# Autophagy is required for PDAC glutamine metabolism

**DOI:** 10.1038/srep37594

**Published:** 2016-11-28

**Authors:** Ju-Won Seo, Jungwon Choi, So-Yeon Lee, Suhyun Sung, Hyun Ju Yoo, Min-Ji Kang, Heesun Cheong, Jaekyoung Son

**Affiliations:** 1 Department of Biomedical Sciences, University of Ulsan College of Medicine, Seoul 138-736, Republic of Korea; 2 Comparative Biomedicine Research Branch, Division of Cancer Biology, National Cancer Center, 323 Ilsan-ro, Ilsandong-gu, Goyang, Gyeonggi-do 410–769, Republic of Korea; 3 Biomedical research center, Department of Convergence medicine, Asan Institute for Life Sciences, Asan Medical Center; 4 Graduate School of Cancer Science & Policy, National Cancer Center, Goyang 410-769, Republic of Korea; 5 Asan Institute for Life Sciences, Asan Medical Center, College of Medicine, University of Ulsan, Seoul, Republic of Korea

## Abstract

Macroautophagy (autophagy) is believed to maintain energy homeostasis by degrading unnecessary cellular components and molecules. Its implication in regulating cancer metabolism recently started to be uncovered. However, the precise roles of autophagy in cancer metabolism are still unclear. Here, we show that autophagy plays a critical role in glutamine metabolism, which is required for tumor survival. Pancreatic ductal adenocarcinoma (PDAC) cells require both autophagy and typical glutamine transporters to maintain intracellular glutamine levels. Glutamine deprivation, but not that of glucose, led to the activation of macropinocytosis-associated autophagy through TFEB induction and translocation into the nucleus. In contrast, glutamine uptake increased as a compensatory response to decreased intracellular glutamine levels upon autophagy inhibition. Moreover, autophagy inhibition and glutamine deprivation did not induce cell death, while glutamine deprivation dramatically activated apoptotic cell death upon autophagy inhibition. Interestingly, the addition of α-ketoglutarate significantly rescued the apoptotic cell death caused by the combination of the inhibition of autophagy with glutamine deprivation. Our data suggest that macropinocytosis-associated autophagy is a critical process providing glutamine for anaplerosis of the TCA cycle in PDAC. Thus, targeting both autophagy and glutamine metabolism to completely block glutamine supply may provide new therapeutic approaches to treat refractory tumors.

Pancreatic ductal adenocarcinoma (PDAC) is one of the most fatal malignancies in humans and continues to be a major medical challenge in the world. It is the seventh leading cause of cancer death worldwide with a 5-year survival rate of 3–5%^1^. Surgery, radiation therapy, and chemotherapy are the treatment options that may extend survival and/or relieve symptoms in many patients; however, pancreatic tumors are highly resistant to cytotoxic chemotherapies, targeted agents, and radiotherapy, which may explain why these treatments are not effective against these tumors^2^. Furthermore, less than 20% of patients are candidates for surgery because pancreatic cancer is usually detected after it has spread beyond the pancreas^3^. Therefore, there is a strong impetus to identify new therapeutic targets and an overwhelming need for new agents to treat this devastating disease.

Unlike normal cells, cancer cells are characterized by increased glycolysis and lactate production regardless of oxygen availability; this is known as the Warburg effect^4^. Proliferating cancer cells exhibit considerably different metabolic requirements compared to most normal differentiated cells. In order to support their high rates of proliferation, cancer cells consume additional nutrients and divert those nutrients into macromolecular synthesis pathways^5^. Metabolic pathways must therefore be rewired in such a way that balances biosynthetic processes with sufficient ATP production to support cell growth and survival. As all cancer cells are dependent on this change in metabolism, these altered pathways represent attractive therapeutic targets.

A number of studies recently demonstrated that many cancers are addicted to glutamine, which can be utilized as a mitochondrial substrate for macromolecular synthesis in cancer cells by providing carbon to fuel the TCA cycle and is the primary nitrogen donor for the synthesis of nucleotides and nonessential amino acids^6^. Glutamine can also generate a significant amount of NADPH through glutaminolysis, including the conversion of glutamine into pyruvate by the malic enzyme. Indeed, PDAC cells maintain cellular redox homeostasis, which is required for cell proliferation by utilizing glutamine for their NADPH needs^7^. Thus, therapies directed against the glutamine metabolism will be most effective in tumors that exhibit glutamine dependence.

Additionally, PDAC cells rely heavily on glutamine for their growth^7^,^8^. However, targeting glutamine metabolism by inhibition of glutaminase significantly reduced PDAC growth, but had no effect on PDAC cell death. This lack of effect on PDAC cell death may be explained by the presence of other metabolic pathways to supply glutamine to the cells. Indeed, it has been reported that cancer cells expressing oncogenic KRas utilize extracellular proteins through macropinocytosis^9^,^10^. A recent study also demonstrated that the extracellular proteins internalized through macropinocytosis serve as a source of glutamine in PDAC^11^. Thus, alternative therapeutic strategies may be developed to target PDAC-specific reliance on glutamine metabolism.

Autophagy is an essential cellular pathway to provide intracellular energy by degradation of unnecessary organelles and macromolecules in response to stimulus such as starvation and accumulation of unfolded proteins^12^. A number of studies indicated important roles of autophagy in cancer. Particularly, there is growing evidence supporting the functional roles of autophagy in cancer metabolism. Autophagy is generally known as a catabolic process that serves to degrade intracellular organelles and recycle their biochemical components to be re-used for energy production and other biosynthetic reactions in conditions of dwindling nutrient supplies and other metabolic perturbations^13^. Thus, in cancer, autophagy is normally induced by limitations in ATP availability^14^,^15^ or a lack of essential nutrients, including glucose^16^ and amino acids^17^,^18^. In contrast, autophagy is elevated in some cancers even in nutrient replete conditions and is required for the growth of these cancers^19^,^20^. It is believed that high levels of autophagy can provide energy as well as an abundance of proteins, sugars, lipids, and nucleic acids, which are required for a rapidly dividing tumor cell. However, the precise roles of autophagy in cancer metabolism are not yet fully understood.

In this study, we demonstrated that pancreatic cancer cells maintain intracellular levels of glutamine via two parallel pathways, including macropinocytosis-associated autophagy and a canonical glutamine transportation pathway. These two parallel pathways control each other to maintain proper intracellular levels of glutamine. In addition, the simultaneous inhibition of both pathways, but not that of each pathway independently, resulted in a pronounced induction of apoptotic cell death, which may provide targeted combination therapies to treat pancreatic cancer.

## Results

### Autophagy is activated upon glutamine deprivation in PDAC

**Figure 1:**
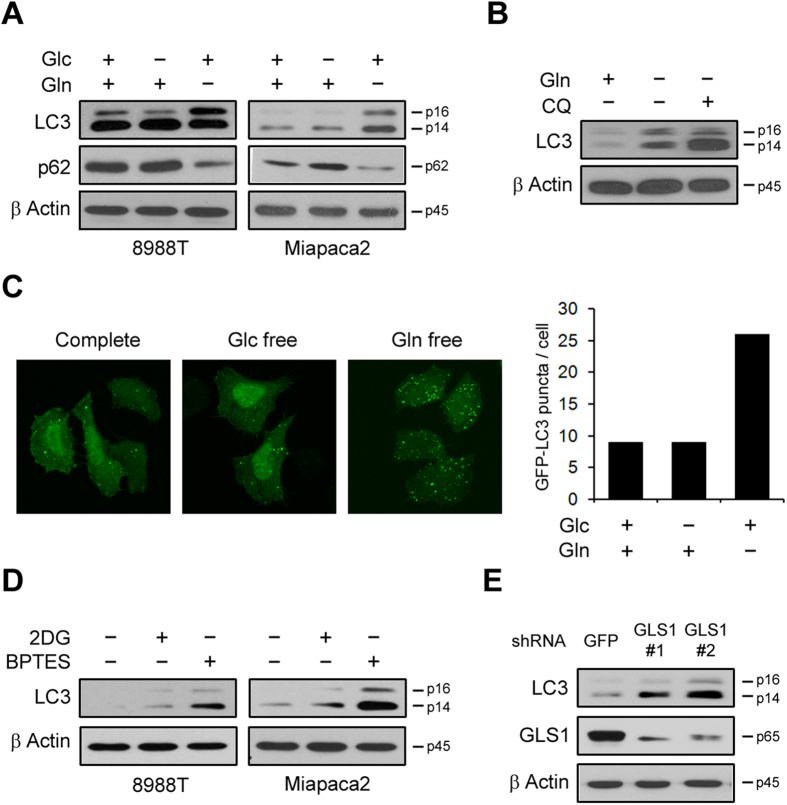
Glutamine, but not glucose, deprivation induces autophagy. (**A**) PDAC cells were plated in complete medium, which was replaced the following day with glucose or glutamine-free medium and then incubated for another 24 h. Cell lysates were immunoblotted for LC3 and p62. (**B**) 8988 T cells were cultured in complete or glutamine free medium with or without CQ (10 μM) for 24 h and immunoblotted for LC3. (**C**) 8988 T cells were infected with a lentivirus expressing GFP-LC3, grown in complete, glucose-free, or glutamine-free medium for 24 h and analyzed for LC3 dots. (**D**) PDAC cells were treated with 2DG (10 mM) or BPTES (10 μM) for 24 h and immunoblotted for LC3. (**E**) Effect of GLS1 knockdown on LC3 levels in 8988 T cells expressing a control shRNA (shGFP) or glutaminase 1 shRNAs (shGLS1s).

Autophagy normally serves as an internal source of nutrients for energy generation during starvation. Thus, it is considered an essential regulator of cellular metabolic homeostasis. However, the precise roles of autophagy in cancer metabolism are not clear. Furthermore, autophagy may have specific functions in cancer metabolism, particularly, in cancers such as pancreatic cancer that present elevated autophagy even in nutrient replete conditions. To explore the specific functional role of autophagy in cancer metabolism, we first investigated the effect of deprivation of either glucose or glutamine, two major nutrient sources for cancer cells, on autophagy in PDAC cell lines. As shown in Fig. 1A, glucose derivation had no significant effect on autophagy activation, whereas glutamine deprivation resulted in a significant increase in LC3-II levels in PDAC cells. Moreover, the levels of p62/A170/SQSTM1 (p62), another marker of autophagy, which is known to be degraded by autophagy^21^, decreased only in glutamine deprivation conditions, indicating that autophagy was only activated in response to glutamine deprivation. Consistent with these results, MEFs expressing a KRas G12V, similar to PDAC that harbors a KRas mutation, showed an increased autophagic flux only upon glutamine deprivation (Fig. S1A). As further evidence for the increase of autophagic flux in response to glutamine deprivation, we treated PDAC cells with 10 μM chloroquine (CQ), a pharmacologic inhibitor of autophagy, which prevents lysosomal acidification, under glutamine deprivation conditions. Inhibition of autophagosome degradation by CQ further accumulated LC3-II levels (Fig. 1B). Furthermore, we examined the recruitment of LC3 into autophagosomes using a GFP-LC3 reporter, which is a standard method to assess activated autophagy^22^. While glucose deprivation had no significant effect on the autophagic flux, the number of GFP-LC3 puncta increased upon glutamine deprivation compared with that in cells cultured in nutrient replete conditions (Fig. 1C). Consistent with these data, treatment with 2-deoxy-d-glucose (2DG), a glucose analog that inhibits glycolysis via its action on hexokinase, had no significant effect on the autophagic flux, whereas levels of LC3-II were dramatically increased in the presence of BPTES, an inhibitor of glutaminase (GLS) (Fig. 1D). In addition to BPTES treatment, GLS knockdown using RNA interference markedly increased LC3-II levels in PDAC cell lines (Fig. 1E and S1B). Glutamine is not an essential amino acid, which means that can be made by the body. Indeed, it can be generated by glutamine synthase. Thus, we next examined whether inhibition of glutamine synthase instead of glutamine deprivation induces autophagy activation. As shown in Supplementary Figure 1C, inhibition of glutamine synthase with methionine sulphoximine had no significant effect on autophagy activation. Thus, these data suggested that the autophagic flux may increase as a compensatory response to glutamine deprivation.

### Autophagy is essential for maintaining intracellular glutamine levels

In addition to glucose, many cancer cells rely on glutamine to meet their biosynthetic needs^23^,^24^. Glutamine is a primary nitrogen donor in nucleotide and amino acid biosynthesis, plays a critical role in maintaining the activation of TOR kinase, and is an important mitochondrial substrate to fuel the TCA cycle^6^. Recent studies reported that many cancer cells utilize glutamine as a key carbon source for the TCA cycle^25^,^26^. Furthermore, glutamine is a primary carbon source for the TCA cycle in the KRas G12D-driven PDAC cells^27^. In addition to glutamine, autophagy is also known as an alternative source to fuel the TCA cycle^13^. Thus, we speculated that autophagy may fuel the TCA cycle via glutamine metabolism because the autophagic flux is induced as a compensatory mechanism only upon glutamine deprivation.

**Figure 2:**
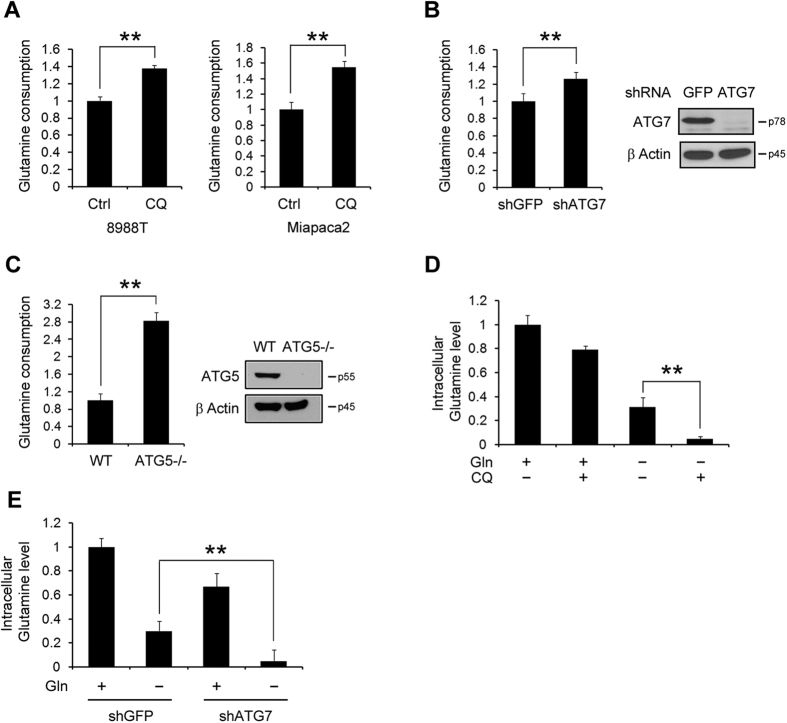
Autophagy is required to maintain intracellular glutamine levels. (**A**) PDAC cells were treated with CQ (10 μM) for 24 h. (**B**) Relative glutamine consumption rate of 8988 T cells expressing a control shRNA (shGFP) or a shRNA targeting ATG7. Western blot confirmed the knockdown of ATG7 expression. (**C**) WT MEFs and *atg5*−/− MEFs expressing KRas G12V were cultured in complete medium for 24 h. Western blot confirmed the knockdown of ATG5 expression. (**A**–**C**) Glutamine levels were measured in the medium using a metabolite analyzer (BioProfile Basic100 analyzer) to monitor glutamine consumption. The glutamine consumption rate was presented after normalization by the number of cells. (**D**) 8988 T cells were plated in complete medium, which was replaced by glutamine-free medium the following day and then incubated for another 24 h with or without CQ (10 μM). Glutamine levels were monitored by using LC-MS/MS. (**E**) 8988 T cells expressing a control (shGFP) or ATG7 shRNA were cultured in complete or glutamine-free medium for 24 h and glutamine levels were monitored by using LC-MS/MS. Error bars represent the s.d. of triplicate wells from a representative experiment. **p < 0.01.

To assess the role of autophagy in glutamine metabolism, we first measured glutamine consumption upon autophagy inhibition by CQ treatment. Interestingly, autophagy inhibition by CQ treatment significantly increased glutamine uptake in PDAC cell lines (Fig. 2A). Consistent with these results, suppression of ATG7 expression by shRNA also induced glutamine consumption (Fig. 2B). Moreover, a mouse model, deficient for autophagy, was utilized to validate the role of autophagy in glutamine metabolism. Consistent with our results in Fig. 2A and B, Ras-expressing *atg5*-knockout (*atg5*−/−) MEFs expressing KRas G12V showed a robust increase in glutamine consumption upon autophagy inhibition when compared to WT MEFs expressing KRas G12V (Fig. 2C). These results indicated that PDAC cells promote glutamine uptake upon autophagy inhibition. Next, we measured intracellular glutamine levels under glutamine deprivation conditions with or without CQ treatment. Interestingly, upon either glutamine deprivation or autophagy inhibition by CQ treatment or ATG7 knockdown, intracellular glutamine levels were partially decreased, while the inhibition of both catabolic pathways further decreased intracellular glutamine levels (Fig. 2D and E). Taken together, our data suggested that two parallel pathways, autophagy and a canonical glutamine transportation pathway may be required to maintain intracellular levels of glutamine in PDAC.

### Glutamine deprivation induces TFEB, which regulates autophagy

**Figure 3:**
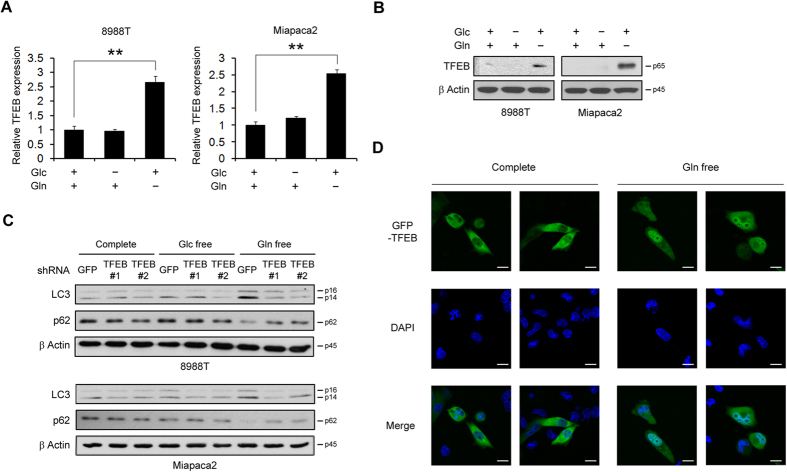
TFEB transcription factor regulates autophagy upon glutamine deprivation. (**A** and **B**) The expression of TFEB was determined by quantitative RT-PCR (**A**) or western blot (**B**) in PDAC cells 24 h after supplementing with glucose or glutamine-free medium. (**C**) PDAC cells expressing a control (shGFP) or TFEB shRNAs (shTFEBs) were plated in the complete medium, which was replaced with glucose or glutamine-free medium the following day and then incubated for another 24 h. Cell lysates were immunoblotted for LC3 and p62. (**D**) Subcellular localization of GFP-TFEB in MIAPaCa2 was monitored by fluorescent microscopy analysis after incubating for 24 hr under either glutamine-replete or glutamine-free conditions, 20 μm. Error bars represent the s.d. of triplicate wells from a representative experiment. **p < 0.01.

We next sought to explore the mechanism by which glutamine deprivation mediates the induction of autophagy. Recent evidence revealed that nutrient deprivation activates a transcription factor EB (TFEB)^28^. TFEB is a main factor for regulating autophagy formation and lysosomal fusion. Thus, cancer cells activate TFEB in response to nutrient deprivation to regulate autophagy, which then degrades unnecessary cellular organelles and macromolecules to provide nutrients. In an effort to determine the mechanism of induction of autophagy upon glutamine deprivation, we measured TFEB transcription levels under complete, glucose or glutamine deprivation. In both normal and glucose deprivation conditions, TFEB mRNA levels were not changed whereas glutamine deprivation resulted in a marked increase in TFEB expression at the mRNA (Fig. 3A) and protein levels in PDAC cells (Fig. 3B). These results demonstrated that glutamine deprivation increases TFEB transcription levels. To confirm the role of TFEB in the regulation of autophagy in response to glutamine deprivation, we detected LC3-II and p62 levels upon TFEB knockdown in glutamine deprivation conditions. As expected, LC3-II levels increased and p62 levels decreased upon knockdown using a control shRNA (shGFP) in the absence of glutamine, while LC3-II and p62 levels were not altered upon TFEB knockdown even when glutamine was depleted (Fig. 3C). The translocation of TFEB into the nucleus as well as the upregulation of TFEB levels is required for TFEB-mediated transcription. Thus, we next investigated whether glutamine deprivation induces the translocation of TFEB into the nucleus. As shown in Fig. 3D, glutamine deprivation clearly promoted TFEB translocation from a cytosolic location to the nucleus. Therefore, these results indicated that the induction of autophagy to compensate intracellular glutamine levels in PDAC cells is mediated by TFEB, which is activated in response to glutamine deprivation.

### Glutamine deprivation by blocking both a canonical glutamine transportation pathway and autophagy exacerbates PDAC cell survival

**Figure 4:**
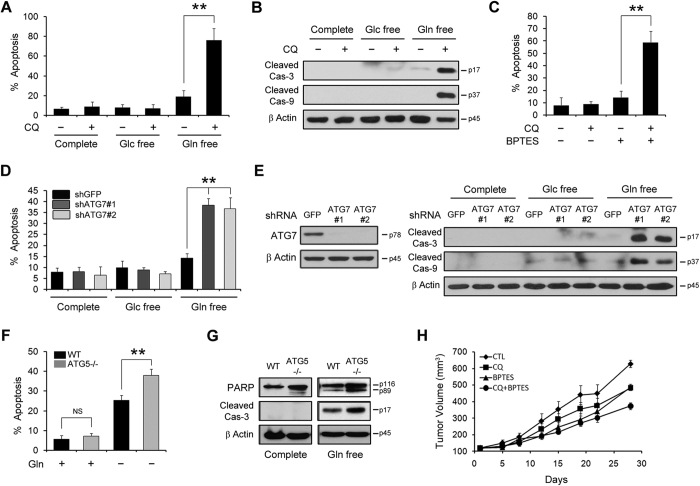
Glutamine deprivation and autophagy inhibition exacerbate Ras mutant cell survival. (**A** and **B**) 8988 T cells were plated in the complete medium, which was replaced with glucose or glutamine-free medium the following day and then incubated for another 24 h with or without CQ (10 μM). Cell death was assessed by using the annexin V/PI assay (**A**) and cleaved caspase-3 and -9 expression was assessed by immunoblotting (**B**). Error bars represent the s.d. of three separate experiments. (**C**) 8988 T cells were treated with CQ (10 μM) and BPTES (10 μM) alone, or in combination for 24 h and cell death was determined by using the annexin V/PI assay. Error bars represent the s.d. of three separate experiments. (**D** and **E**) 8988 T cells expressing a control (shGFP) or ATG7 shRNAs (shATGs) were plated in the complete medium, which was replaced with glucose or glutamine-free medium the following day and then incubated for another 24 h. Cell death was assessed by using the annexin V/PI assay (**D**) and cleaved caspase-3 and -9 expression was assessed by immunoblotting. ATG7 knockdown was confirmed by western blot (**E**). Error bars represent the s.d. of three separate experiments. (**F** and **G**) WT MEFs and *atg5*−/− MEFs expressing KRas G12V were plated in the complete medium, which was replaced with glutamine-free medium the following day and then incubated for another 24 h. Cell death was assessed by using the annexin V/PI assay (**F**) and immunoblotted for PARP and cleaved caspase-3 expression was assessed by immunoblotting (**G**). Error bars represent the s.d. of three separate experiments. (**H**) Subcutaneous MIAPaCa2-driven tumors were established in 6-week old male mice. CQ (20 mg kg^−1^ per day), BPTES (4 mg kg^−1^ per day) alone, or in combination, were administered daily via intraperitoneal injection. Tumor growth was assessed once the tumor volume reached 150 mm^3^. Data are shown as the mean of five mice in each group ± SEM. NS, not significant. **p < 0.01.

It has been reported that basal autophagy is required for PDAC growth^19^,^20^ and glutamine is a crucial nutrient for PDAC growth^7^. These groups showed that the suppression of either autophagy or glutamine deprivation significantly impaired PDAC growth, but the effect of either autophagy inhibition or glutamine withdrawal on PDAC cell death was not demonstrated. Our data indicated that inhibition of one pathway, either autophagy or glutamine metabolism, upregulated the other pathway as a compensatory mechanism and both autophagy and a canonical glutamine transportation pathway are indispensable to maintain intracellular glutamine levels. Therefore, we hypothesized that glutamine deprivation with autophagy inhibition may lead to PDAC cell death via almost complete depletion of intracellular glutamine levels. Indeed, either suppression of autophagy or glutamine deprivation had no effect on apoptotic cell death, whereas apoptotic cell death was dramatically induced upon glutamine deprivation with CQ treatment (Fig. 4A). Consistent with these results, caspase-3 and caspase-9 were robustly activated only when glutamine was depleted by CQ treatment (Fig. 4B). Moreover, BPTES treatment activated apoptosis only upon CQ treatment in PDAC cells (Fig. 4C and S2A). To confirm the effect of autophagy inhibition and glutamine deprivation on PDAC cell death, we impaired ATG7 expression using two lentiviral shRNAs in PDAC cells. ATG7 knockdown induced apoptosis more effectively under glutamine deprivation conditions (Fig. 4D). Consistently, ATG7 knockdown resulted in the robust activation of caspase-3 and caspase-9 only upon glutamine deprivation (Fig. 4E). Moreover, glutamine deprivation induced apoptotic cell death in Ras-expressing *atg5*−/− MEFs and apoptotic cell death was further activated in Ras-expressing *atg5*−/− MEFs compared with that in WT MEFs (Fig. 4F and G).

We next sought to investigated whether glutamine deprivation with autophagy inhibition is a potential therapeutic strategy for PDAC treatment *in vitro* and *in vivo*. We first investigated the effect of the combination of BPTES plus CQ on PDAC growth *in vitro*. Either BPTES or CQ treatment for 72 h had no significant effect on PDAC growth whereas the combination of BPTES plus CQ resulted in a marked decrease in PDAC growth (Fig. S2B). Further, to confirm the importance of both a canonical glutamine transportation pathway and autophagy in PDAC growth, we assessed their ability to grow *in vivo* as xenografts. As shown in Fig. 4H, treatment with BPTES or CQ alone had a modest effect on tumor growth. In contrast, the combination of BPTES plus CQ effectively inhibited tumor growth. Therefore, *in vitro* and *in vivo* data further support the essential role of distinct metabolic processes to supply glutamine for PDAC growth.

### Macropinocytosis controls intracellular glutamine levels through autophagy

**Figure 5:**
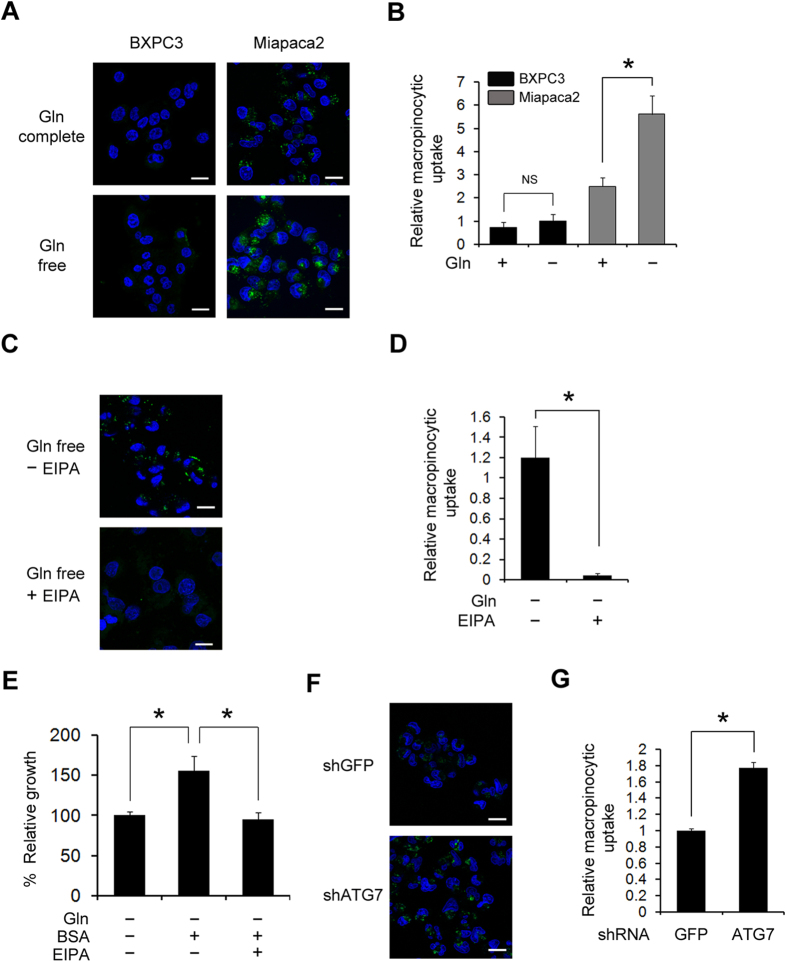
Glutamine deprivation increases macropinocytosis (**A**) Macropinocytosis was assessed in BxPC3 and MIAPaCa2 cells by monitoring the uptake of FITC-Dextran under either glutamine-replete or glutamine-free conditions. Scale bars, 20 μm. (**B**) The relative macropinocytic uptake of BxPC3 and MIAPaCa2 were quantified by image-based determination of the total macropinocytic vesicle area compared with the DAPI-stained area of cells. Data are expressed relative to the values of BxPC3 observed in the glutamine-free conditions. Data are shown as the mean of five images in each experiment ± SEM. (Error bars represent the SEM of values from five representative images per experiment) (**C**) Macropinocytic uptake of MIAPaCa2 cells induced by glutamine deprivation was inhibited by the treatment with EIPA (50 μM). (**D**) The levels of macropinocytic uptake of MIAPaCa2. MIAPaCa2 were quantified by image-based determination as shown in (**B**). Data are expressed relative to the values of MIAPaCa2 in the glutamine-free conditions. (**E**) MIAPaCa2 cells were cultured in glutamine deprivation medium for 6 days, either with or without 2% BSA and further with EIPA treatment. Growth levels were measured by cell counting. Data are expressed relative to the values observed in the glutamine-free conditions. (**F**) MIAPaCa2 cells expressing ATG7 shRNA and control shRNA were cultured in glutamine-free medium for 16 h after treatment with FITC-Dextran and macropinocytic degradation was determined by monitoring intracellular FITC-Dextran. Scale bars, 20 μm. (**G**) Levels of macropinocytic uptake of MIAPaCa2 cells were quantified by image-based determination as shown in (**B**). Data are relative to the values of MIAPaCa2 cells cultured in glutamine-free conditions. Error bars represent the SEM of triplicates from a representative experiment. *p < 0.05.

Our data revealed that PDAC cells require autophagy to replenish glutamine for their growth. However, the precise mechanism by which autophagy provides glutamine remains unclear. To address this question, we next sought to explore the mechanism by which autophagy provides glutamine to PDAC cells. Based on our previous results indicating that BSA uptake in Ras-expressing cells via macropinocytosis supports cell growth under glutamine-deprived conditions^29^, we examined whether glutamine deprivation directly regulates macropinocytosis in addition to autophagy. Recent studies reported that macropinocytosis contributes to cell growth and survival in cancer cells through the maintenance of the nutrient balance^9^,^11^,^30^. Macropinocytosis is a regulated endocytic pathway that accompanies cell surface ruffling and internalizes extracellular proteins and associated components through macropinosome. Following degradation, the internalized proteins fulfill cell metabolic requirements. Ras-transformed cells induce macropinocytosis to meet the demand for increased metabolites needed to support rapid growth of cancers. More interestingly, oncogenic Ras-induced macropinocytosis supplies amino acids, particularly, glutamine, and KRas is the most frequently mutated gene in PDAC^11^. Thus, we hypothesized that oncogenic Ras-induced macropinocytosis requires autophagy to degrade extracellular proteins, which results in a sufficient supply of glutamine in PDAC cells. To investigate the effect of macropinocytosis on autophagy-mediated glutamine supply, we performed fluorescence microscopy analysis to monitor the intracellular uptake of dextran as a marker of macropinocytosis. Indeed, glutamine deprivation led to a robust increase in macropinocytosis only in MIAPaCa2, which harbors a KRas mutation (Fig. 5A and B). The increase in macropinocytosis upon glutamine deprivation was significantly inhibited by 5-(*N*-ethyl-*N*-isopropyl)-amiloride (EIPA), which is known as a macropinocytosis inhibitor^31^ (Fig. 5C and D). To further confirm the role of macropinocytosis in supplying glutamine, we next treated cells grown in glutamine-free conditions with extracellular BSA. As shown in Fig. 5E, BSA supplementation significantly enhanced the relative growth when compared to cell growth upon glutamine deprivation conditions. Furthermore, this pro-proliferative effect was inhibited by EIPA treatment.

Macropinocytosis requires degradation of extracellular proteins to supply glutamine to the cell. A recent study showed that autophagy inhibition by bafilomycin A1 treatment inhibited oncogenic Ras-mediated macropinocytosis^11^. Additionally, through macropinocytosis, the internalized proteins dramatically accumulated in Ras-expressing *atg5*−/− MEFs when compared with WT MEFs^29^. Thus, we next examined whether autophagy is necessary for macropinocytosis-mediated glutamine supply. Consistent with our previous studies, ATG7 knockdown significantly increased macropinocytic uptake under glutamine-free conditions (Fig. 5F and G), indicating that autophagy could play a critical role in the degradation of internalized macromolecule associated with macropinocytosis to supply glutamine in PDAC cells.

### Inhibition of macropinocytosis-mediated glutamine supply upon glutamine deprivation in PDAC induces apoptotic cell death via depleting TCA cycle intermediates

**Figure 6:**
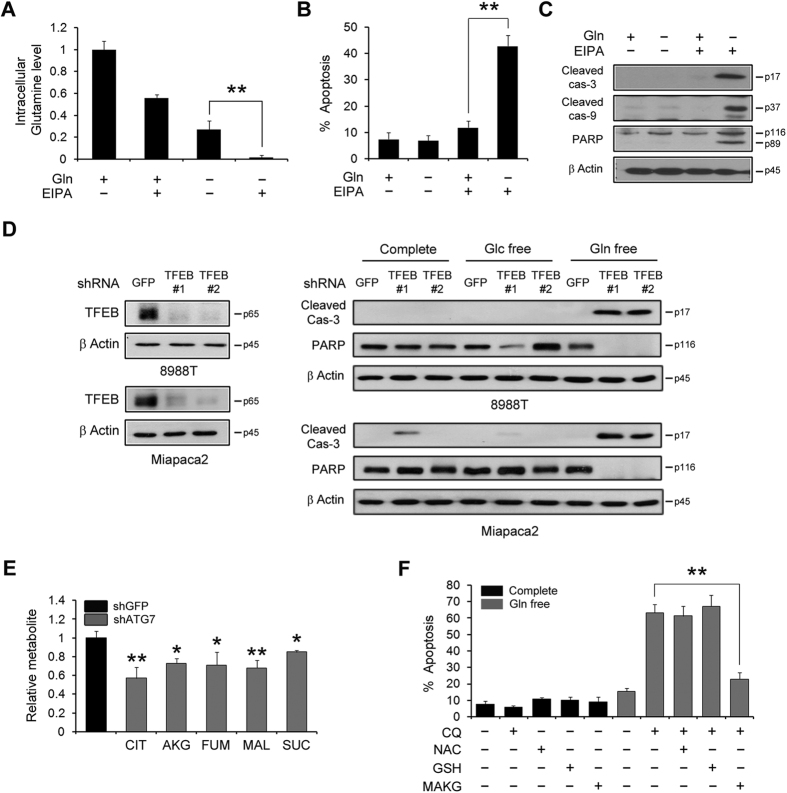
Inhibition of metabolic pathways providing substrates for the TCA cycle induces apoptotic cell death in PDAC. (**A**) 8988Tcells were plated in the complete medium, which was replaced by glutamine-free medium the following day and then incubated for another 24 h with or without EIPA (25 μM). Glutamine levels were monitored by using LC-MS/MS. Error bars represent the s.d. of triplicate wells from a representative experiment. (**B** and **C**) PDAC cells were plated in the complete medium which was replaced by glutamine-free medium the following day and then incubated for another 24 h with or without EIPA (25 μM) for 24 h. Cell death was assessed by using the annexin V/PI assay (**B**) and cleaved-3, -9 and PARP expression was determined by immunoblotting (**C**). Error bars represent the s.d. of three separate experiments. (**D**) 8988 T cells expressing a control shRNA (shGFP) or TFEB shRNAs (shTFEBs) were plated in the complete medium, which was replaced by glucose or glutamine-free medium the following day and then incubated for another 24 h. Cell lysates were immunoblotted for cleaved caspase-3 and PARP. TFEB knockdown was confirmed by western blot. (**E**) TCA metabolite pools were analyzed by using LC-MS/MS in 8988 T cells expressing a control (shGFP) or ATG7 shRNA. Error bars represent the s.d. of triplicate wells from a representative experiment. (**F**) 8988 T cells were plated in the complete medium, which was replaced by glutamine-free medium supplemented with NAC (2 mM), GSH (2 mM), or MAKG (2 mM) in the absence or presence of CQ (10 μM) the following day and then incubated for another 24 h. NAC, *N*-acetylcysteine; GSH, glutathione; MAKG, dimethyl α-ketoglutarate. Error bars represent the s.d. of triplicate wells from a representative experiment. *p < 0.05; **p < 0.01.

Given that intracellular glutamine levels were almost exhausted upon glutamine deprivation with autophagy inhibition, leading to apoptosis, we examined whether inhibition of macropinocytosis upon glutamine deprivation reduces intracellular glutamine levels. Consistent with our previous results presented in Fig. 2D, intracellular glutamine levels were decreased upon glutamine deprivation. When combined with EIPA, this effect was dramatically augmented (Fig. 6A), which resulted in the activation of apoptosis (Fig. 6B and C) and the combination of BPTES plus EIPA had a significant effect on the activation of apoptosis (Fig. S3A and B). Knockdown of Rac1, which is required for macropinocytosis, also activated apoptosis only upon low glutamine condition (Fig. S3C). Furthermore, unlike PDAC cells, BxPC3 cells which did not exhibit an increase in macropinocytosis when placed in glutamine-free media (Fig. 5A) did not undergo apoptosis upon inhibition of glutamine metabolism and autophagy (Fig. S3D and E). The combination of CQ plus amiloride (AM; a macropinocytosis inhibitor) had no additive effect on the inhibition of tumor growth (Fig. S3F). Thus, these data indicate that both macropinocytosis and typical glutamine transporters are critical to maintain intracellular glutamine levels for PDAC survival.

In addition to the role of macropinocytosis for glutamine supply, we showed that TFEB mediates autophagy activation upon glutamine deprivation in Fig. 3. Thus, we next tested whether TFEB, required to maintain intracellular glutamine levels, is also necessary to support PDAC survival. TFEB knockdown resulted in a decrease in Cathepsin K levels (Fig. S4), indicating that TFEB has indeed impacted lysosomal biogenesis. TFEB knockdown markedly cleaved both caspase-3 and PARP in a manner similar to that of ATG7 knockdown (Fig. 6D), suggesting that PDAC requires both autophagy and a canonical glutamine transportation pathway for survival.

**Figure 7:**
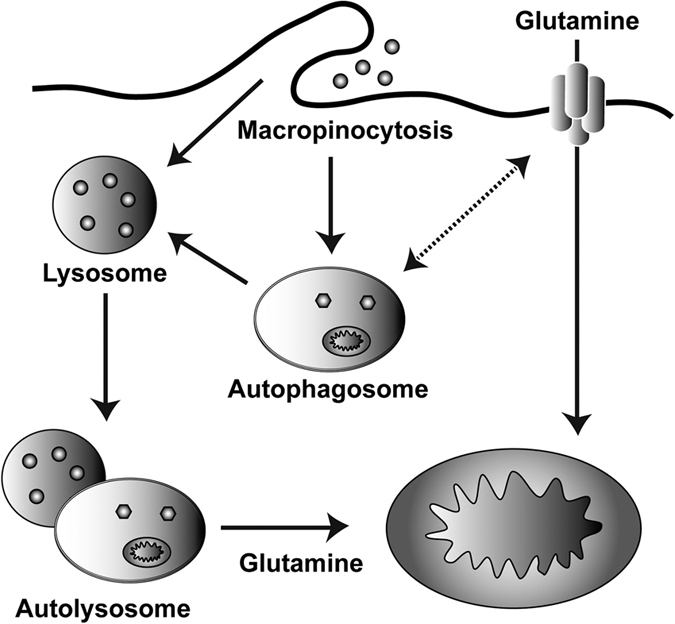
Model of maintaining intracellular levels of glutamine via two parallel pathways, including macropinocytosis-associated autophagy and a canonical glutamine transportation pathway.

We next sought to explore the mechanism by which the inhibition of macropinocytosis-mediated glutamine supply via autophagy upon glutamine deprivation triggers apoptosis. To this end, we first measured intermediate levels of the TCA cycle upon inhibition of autophagy with either ATG7 knockdown or CQ treatment. Inhibition of autophagy resulted in a marked decrease in the intermediate levels of the TCA cycle (Fig. 6E and S5A). To confirm the role of glutamine metabolism in PDAC cell death, we next sought to rescue apoptotic cell death with anti-oxidants, including *N*-acetyl-l-cysteine (NAC) and Glutathione (GSH) or an intermediate of the TCA cycle, alpha-ketoglutarate (α-KG). Interestingly, antioxidants did not rescue cell death caused by inhibition of both autophagy and glutamine metabolism, whereas α-KG was able to robustly rescue KRas-driven cancer cells from death (Fig. 6F and S5B). Consistent with this observation, α-KG largely rescued cells from glutamine deprivation-induced cell death in Ras-expressing *atg5*−/− MEFs (Fig. S5C). Additionally, the apoptotic cell death caused by glutamine deprivation with EIPA treatment was restored to almost normal levels in the presence of α-KG (Fig. S6A and B). Therefore, our data indicated that two parallel pathways, autophagy and a canonical glutamine transportation pathway, are necessary to maintain intracellular glutamine levels required for PDAC survival by supporting the TCA cycle (Fig. 7).

## Discussion

Cancer cells have two major strategies for rapid and continuous proliferation. The first strategy is to meet the bioenergetic and biosynthetic demands of increased cell proliferation. Metabolic pathways must therefore be rewired in order to meet the increased requirements for proliferation. The second strategy is to survive in a nutrient-limited environment that defines most cancers. Cancer cells normally live in an environment with a limited nutrient supply for their proliferation. However, they are still able to survive in these conditions. Therefore, as far as metabolic aspects are concerned, rapidly growing cancer cells require an alternative metabolic pathway, allowing them to get an abundance of proteins, sugars, lipids, and nucleic acids even under nutrient-limited conditions.

Autophagy captures large structures such as protein aggregates and organelles and degrades them in the lysosomes. The resulting breakdown products into the cytoplasm are reused to reuse energy, proteins, and membranes. In general, cancer cells activate the autophagic flux, which leads to recycling of intracellular components into metabolic pathways to maintain metabolism and energy homeostasis during starvation. Maintaining metabolic homeostasis as a compensatory mechanism in nutrient-limited conditions is an essential role of autophagy. In contrast, PDAC cells have evolved to require high levels of autophagy for growth even under nutrient complete conditions for tumor maintenance^19^,^20^. These studies demonstrate that PDAC cells require autophagy as an alternative metabolic pathway to supply an abundance of substrates for mitochondrial metabolism. Cancer cells utilize a variety of nutrient sources to fuel TCA cycle^5^, among which glutamine is replenished into the TCA cycle after conversion to the α-KG in many cancer cells^32^,^33^. In PDAC cells, glutamine is utilized as the main carbon source for the TCA cycle^27^. In this study, we demonstrated that autophagy is essential for glutamine supply, which is utilized to maintain pools of TCA cycle intermediates. Autophagy inhibition led to a profound reduction in intracellular glutamine levels. Similar results were observed in *atg5*−/− MEFs. Thus, our data indicate that both autophagy and the typical glutamine uptake by transporters are critical to maintain intracellular glutamine levels. These results are also supported by the observation that glutamine deprivation with autophagy inhibition resulted in the almost complete depletion of intracellular glutamine levels.

TFEB is the member of the MiTF/TFE family identified as a master regulator of lysosomal biogenesis and also induce the expression of a subset of autophagy, which are critical regulators of autophagosome formation^34^. TFEB is localized largely in the cytosol in nutrient replete conditions. In contrast, following starvation TFEB expression is increased and it rapidly translocates from the cytosol to the nucleus, leading to autophagy activation in order to survive under the nutrient deprivation conditions^35^,^36^. Interestingly, we showed that deprivation of glucose, one of the major nutrients, did not activate the autophagic flux in PDAC cells. However, glutamine deprivation resulted in a significant increase of TFEB levels and its translocation into the nucleus, leading to LC3-II accumulation and autophagosome formation. mTORC1 is known to induce translocation of TFEB into the nucleus. Thus, it is believed that glutamine deprivation-activated TFEB expression and translocation into the nucleus is mTORC1-associated mechanism, given that amino acid, glutamine in particular had been causally linked to amino acid-dependent mTORC1 activation^37^,^38^. However, further work must be performed to determine the mechanisms whereby glutamine deprivation induces TFEB activation and translocation into the nucleus.

Previous studies suggested that autophagy is important for glutamine metabolism. Autophagy deficiency impaired mitochondrial metabolism, which resulted in the inhibition of tumor growth and the addition of glutamine or pyruvate, but not that of glucose or ROS scavenger, rescued the survival of atg7-deficient tumor-derived cell lines^39^. Additionally, autophagy was activated upon glutamine deprivation and reversed by glutamine supplementation^40^. However, the precise role of autophagy in glutamine metabolism was not fully understood yet. Here, we provided additional evidence that autophagy is a process providing glutamine for supporting anaplerosis of the TCA cycle. Moreover, oncogenic KRas-expressing pancreatic cancer cells showed high levels of basal macropinocytosis to utilize extracellular proteins for tumor growth, which is closely associated with autophagy^11^,^29^. Our study showed that additional deprivation in glutamine as well as serum further increases macropinocytosis levels when compared to that in serum starvation alone. This phenotype was inhibited by a macropinocytosis inhibitor in MIAPaCa2 cells which harbor a KRas mutation, but not in BXPC3 cells which harbors a KRas WT. Furthermore, macropinocytic degradation was inhibited upon ATG7 knockdown and severe accumulation of macropinocytic markers was observed, which is consistent with the effect of glutamine deprivation with autophagy inhibition on intracellular glutamine levels. Both glutamine deprivation and macropinocytosis inhibition also led to almost complete depletion of intracellular glutamine levels.

Glutamine is an essential nutrient for proliferating cancer cells. In particular, KRas-transformed cells exhibit an increased dependency on glutamine for growth^41^,^42^ and a recent study demonstrated that glutamine deprivation profoundly impairs PDAC growth^7^, indicating that glutamine is critical for PDAC growth. In addition, autophagy contributes to the growth and survival of KRas-transformed cancers such as PDAC^19^,^20^ and during reviewing our manuscript, White group has also reported that autophagy supplies substrates to maintain levels of amino acid, practically glutamine, in Ras-driven lung cancer cells^43^. However, the precise connection between glutamine supply and autophagy and the effect of both processes on cell death are not fully understood. Our results indicate that autophagy inhibition conversely enhanced canonical glutamine uptake to compensate the lack of intracellular levels of glutamine. Moreover, glutamine deprivation with autophagy inhibition led to a robust activation of apoptotic cell death, whereas either glutamine deprivation or autophagy inhibition alone had no significant effect on apoptotic cell death due to upregulation of other processes to maintain intracellular glutamine levels as a compensatory mechanism. Most importantly, the inhibition of any step in macropinocytosis-associated autophagy for glutamine supply markedly induced apoptotic cell death upon glutamine deprivation. Additionally, our data revealed that apoptotic cell death caused by glutamine deprivation with the inhibition of autophagy was restored to almost normal levels in the presence of α-KG, further supporting the importance of glutamine as a key carbon source for the TCA cycle and for PDAC survival.

In summary, we propose a unique mechanism in which autophagy plays a critical role in PDAC glutamine metabolism. Autophagy is highly activated even under normal conditions for macropinocytosis-associated glutamine supply in PDAC to maintain an abundant supply of substrates for mitochondrial metabolism. Thus, autophagy is an attractive therapeutic target in KRas-driven tumors. In fact, CQ and other autophagy inhibitors have been studied as anti-tumor drugs, although the precise mechanism by which CQ exerts its anti-tumor effects is not clear. Based on our findings, concomitant targeting the glutamine metabolism and autophagy (or macropinocytosis) would provide an appropriate therapeutic rationale for KRas-driven tumors, including PDAC.

## Materials and Methods

### Chemicals and reagents

Primary antibodies against LC3B, ATG7, PARP, cleaved caspase-3, and cleaved caspase-9 were purchased from Cell Signaling Technology (Beverly, MA, USA). The primary antibody against p62 was purchased from Progen (Heidelberg, Germany), β-actin from Sigma-Aldrich (St Louis, MO, USA), and Cathepsin K and tubulin from Santa Cruz Biotechnology (Dallas, Texas, USA). The GLS antibody was obtained from Abcam (Cambridge, MA, USA) and the antibody against TFEB was purchased from Bethyl Laboratories (Montgomery, TX, USA). The secondary antibodies, horseradish peroxidase (HRP)‐conjugated anti‐rabbit and anti‐mouse antibodies were from Bethyl Laboratories. Non‐essential amino acids (1567906), essential amino acids (1542383), glutamine (25030081), HEPES (1627660), vitamin solution (1567870), and sodium bicarbonate (1546264) were from Life Technologies (Carlsbad, CA, USA). DAPI, Hoechst 33258, fluorescein-conjugated albumin from bovine serum (BSA: A23015), Dextran, fluorescein (10 KDa; D1820 were from Life Technologies (Carlsbad, CA, USA). Mounting media (S3023) was from DAKO (Carpinteria, CA, USA). Bovine serum albumin (BSA; A1470), CQ (C6628), amiloride (A7410), 5-(*N*-ethyl-*N*-isopropyl) amiloride (EIPA; A3085), phosphatase inhibitor cocktail, BPTES (SML0601), dimethyl α-KG (349631), *N*-acetyl-l-cysteine (NAC; A9165), and GSH reduced ethyl ester were from Sigma-Aldrich. Protease inhibitor cocktail tablets were from Roche Applied Bioscience (Basel, Switzerland).

### Cell lines and culture conditions

The 8988 T, BxPC3, and MIAPaCa2 human pancreatic cancer cell lines from the American Type Culture Collection (ATCC; Manassas, VA, USA) were kindly provided by Yun-Hee Kim (National Cancer Center, Korea). ATG5 KO MEFs were generated by mating ATG5 heterozygous mice generously provided by Dr. Noboru Mizushima (Tokyo Medical and Dental University, Bunkyo-Ku, Japan)^44^. All cells were maintained in a 5% CO_2_ atmosphere at 37 °C in medium supplemented with 10% FBS (Hyclone), 100 U/mL penicillin, and 100 μg/mL streptomycin (Life Technologies). MEFs, 8988 T, and MIAPaCa2 cells were maintained in Dulbecco’s modified Eagle’s medium (DMEM; Life technologies). BXPC3 were cultured in RPMI1640 (Life technologies). For either glucose or glutamine starvation, DMEM without glucose (Sigma-Aldrich) or DMEM without glutamine (Life Technologies) was supplemented with 10% dialyzed-FBS (Life technologies). Constitutively activated Ras GTPase plasmids were from Addgene. pBabe K-Ras G12V (#9052) was generously provided by William Hahn through Addgene. Oncogenic Ras-expressing MEFs were generated following standard protocols for retrovirus transduction.

### Western blotting

For western blotting, cells were rinsed with ice‐cold PBS and harvested using ice‐cold lysis buffer (RIPA buffer). Samples were then incubated in RIPA buffer (50 mM TRIS-Cl pH 7.4, 150 mM NaCl, 1% NP-40, 0.5% Na-deoxycholate, 0.1% SDS, 1 mM EDTA) with protease inhibitor cocktail (Roche Applied Bioscience) and phosphatase inhibitor (Sigma-Aldrich) for 15 min and soluble lysate fractions were isolated by centrifugation at 16,000× *g* for 15 min. Protein concentrations were determined with the Pierce BCA Protein Assay (Thermo Scientific, Waltham, MA, USA) and equal amounts of protein were analyzed by SDS gel electrophoresis and western blotting following standard protocols.

### Fluorescence microscopy

For imaging of macropinocytosis, cells were starved (no FBS) for 4 h to increase macropinocytosis and the medium was then changed to either glutamine complete or glutamine free medium in addition to serum starvation. These cells were incubated with 0.5 mg/mL dextran or BSA conjugated with FITC or TMR for the indicated periods at 37 °C. Subsequently, cells were washed 3 times with ice-cold PBS and fixed with 3.7% formaldehyde in PBS for 15 min. Nuclei were then stained using DAPI and mounted using mounting media (DAKO). Images were captured using an LSM780 confocal fluorescent microscope (Zeiss) and quantified using the Image J (National Institutes of Health, Bethesda, MD, USA). The total macropinosome area per cell was normalized by the area of DAPI stained nucleus of that cell. Macropinosome areas were quantified in at least five distinct fields captured from different regions for an individual experimental set. Subcellular localization of GFP-TFEB or GFP-LC3 was monitored by either LSM780 confocal fluorescent microscope (Carl Zeiss, Germany) or Axio Observer Z1 fluorescence microscope (Carl Zeiss, Germany), respectively.

### Glutamine measurement

Glutamine consumption was measured using the BioProfile analyzer (Nova Biomedical, MA, USA). Briefly, cells were plated in 6-well plates in complete medium, which was replaced by glutamine-free medium the following day and then incubated for another 24 h with or without reagents. Glutamine concentration in the media was then measured and normalized to the number of cells in each well. The controls were plated with an equal volume of medium but no cells, incubated identically as the cell-containing plates.

### Cell growth and viability

Cells were plated in complete media on either 96-well or 24-well plates prior to starvation. Twenty-four hours after seeding, cells were washed with PBS and incubated in the indicated glutamine starvation medium with 10% dialyzed FBS. For rescue experiments, cells were incubated in the medium supplemented with 2% BSA. Negative control cells for macropinocytosis were treated with EIPA. The number of cells was counted either using a Coulter Counter (Beckman Coulter). Cell viability was determined by annexin-V/FITC assay. Cells were harvested by trypsinization and washed with PBS, and resuspended in annexin-V binding buffer (10 mM HEPES, pH 7.4, 140 mM NaCl, 2.5 mM CaCl2) containing annexin-V fluorescein isothiocyanate (FITC) and propidium iodide (PI). Stained cells were quantified and analyzed by using a flow cytometer (Beckman-Coulter).

### Mouse xenograft studies

Six-week-old female BALB/C nude mice (Orient Bio Inc., Korea) were handled using aseptic procedures and allowed to adjust to local conditions for 1 week before experimental manipulations began. MIAPaCa2 cells (5 × 10^6^) were mixed at a 1:1 dilution with matrigel (BD Biosciences, Franklin Lakes, NJ, USA) and injected subcutaneously into both flanks of each mouse for a total final volume of 200 μL. When tumors reached an average volume of 150 mm^3^, BPTES (4 mg kg^−1^ per day), CQ (20 mg kg^−1^ per day) alone, or a combination of both were administered daily via intraperitoneal injection for up to 33 days. Tumor growth was evaluated by measurement of two perpendicular diameters of the tumors and tumor size was calculated using the formula 4π/3 × (width/2)^2^ × (length/2). The tumors were harvested and weighed at the experimental endpoint. Animal experiments were performed in accordance with protocols approved by the Institutional Animal Care and Use Committee at the National Cancer Center, Republic of Korea. The methods applied in this study were performed in accordance with the approved guidelines.

### Metabolomics

Cells were grown to about 60% confluence in growth medium (DMEM, 10% FBS) on 10-cm dishes in biological triplicate. After 24 h, the cells were then harvested using 1.4 mL of cold methanol/H2O (80/20, v/v) after sequential wash with PBS and H_2_O and lysed by vigorous vortexing and 100 μL of 5 μM of internal standard was added. Metabolites were extracted from the aqueous phase by liquid-liquid extraction after adding chloroform. The aqueous phase was dried using vacuum centrifuge and the sample was reconstituted with 50 μL of 50% methanol prior to LC-MS/MS analysis. The LC/MS/MS system was equipped with an Agilent 1290 HPLC (Agilent), Qtrap 5500 (ABSciex), and reverse phase column (Synergi fusion RP 50 × 2 mm). Three microliters were injected into the LC-MS/MS system and ionized with a turbo spray ionization source. Multiple reaction monitoring (MRM) was used in the negative ion mode and the extracted ion chromatogram (EIC) corresponding to the specific transition for each metabolite was used for quantitation. Area under the curve of each EIC was normalized to that of EIC of internal standard. The peak area ratio of each metabolite to that of the internal standard was normalized using the protein amount in each sample and was then used for relative comparison.

### Quantitative real-time PCR

Total RNA was isolated using TRIzol (QIAGEN, Hilden Germany) and cDNA was synthesized using 2 μg of total RNA using oligo-dT and MMLV HP reverse transcriptase (Epicentre). Quantitative real time PCR was performed with SYBR Green dye using an AriaMx Real-Time PCR System. The relative amount of cDNA was calculated by the comparative Ct method using the 18 S ribosomal RNA sequences as a control. The primer sequences were designed as follows: *SLC1A1* (forward: GCGAGGAAAGGATGCGAGT, reverse: GCTGTGTTCTCGAACCAAGACT), *SLC1A2* (forward: TGTCCACGACCATCATTGCTG, reverse: TTCTTGAGCTTGGGATTGCCT), *SLC1A3* (forward: AGCAGGGAGTCCGTAAACG, reverse: AGCATTCCGAAACAGGTAACTTT), *SLC1A5* (forward: CCCTCATCTACTTCCTCTTCAC, reverse: TTATTCTCCTCCACGCACTTC), *SLC1A6* (forward: AGCAGCCCACACATCTCATC, reverse: GTGTTCCCAGTTTCTACAGGGT). *SLC7A5* (forward: GTGGACTTCGGGAACTATCACC, reverse: GAACAGGGACCCATTGACGG), *SLC7A6* (forward: CTCTTACTCAGGTTGGGACAC, reverse: TTCAGCACTGTGTAATAGGCC), *SLC7A8* (forward: CTATGGAGGCTGGAACTTTCTG, reverse: GCAGTGACATAAGCGACATTG), SLC38A1 (forward: TGACAGTGCCCGAGGATGATA, reverse: AGACATGCCTAAGGAGGTTGTA), and *SLC38A2* (forward: ACCGCAGCCGTAGAAGAATG, reverse: GCCAGACGGACAATGAGAAGAA). The *TFEB* primer sequence was as follow: *TFEB* forward: GAGAATCCCACATCCTACCATC, reverse: GCAGCAAACTTGTTCCCATAG.

### Autophagy assay

To measure the autophagic flux, PDAC cells were infected with a virus encoding GFP-LC3 and plated in 12-well plates. Before plating, the plate was washed 3 times with DPBS and covered with a round cover glass coated with a factor to allow cell adhesion. For nutrient deprivation, cells were plated in complete culture medium (10 mM glucose and 2 mM glutamine), which was replaced by glutamine-free medium the following day. Cells were fixed in 4% paraformaldehyde and then transferred to a microscope slide glass (MARIENFELD) and GFP-LC3 was observed through the inverted microscope equipment and analyzed by using the ZEN 2012 program.

### Lentiviral-mediated shRNA and siRNA

The RNAi Consortium clone IDs for the shRNAs used in this study are as follows: TRCN0000051135 (shGLS-1), TRCN0000051136 (shGLS-2), TRCN0000007584(shATG7-1), TRCN0000007587 (shATG7-2), TRCN0000013109 (shTFEB-1), and TRCN0000013112 (shTFEB-2). Rac1 siRNA was purchased from Genolution, target sequence: GAGGCCUCAAGACAGUGUUUGACGA.

## Additional Information

**How to cite this article**: Seo, J.-W. *et al.* Autophagy is required for PDAC glutamine metabolism. *Sci. Rep.* **6**, 37594; doi: 10.1038/srep37594 (2016).

**Publisher's note:** Springer Nature remains neutral with regard to jurisdictional claims in published maps and institutional affiliations.

## Supplementary Material

Supplementary Information
