# Supplementary material for: Autophagy is required for PDAC glutamine metabolism
Source: Sci Rep. 2016 Nov 28;6:37594. doi: 10.1038/srep37594 (PMC5124864; doi:10.1038/srep37594)
Supplement: Supplementary Information [file srep37594-s1.pdf]

Supplementary Information

Autophagy is required for PDAC glutamine metabolism

Ju-Won Seo, Jungwon Choi, So-Yeon Lee, Suhyun Sung, Hyun Ju Yoo, Min-Ji Kang, Heesun Cheong, Jaekyoung Son

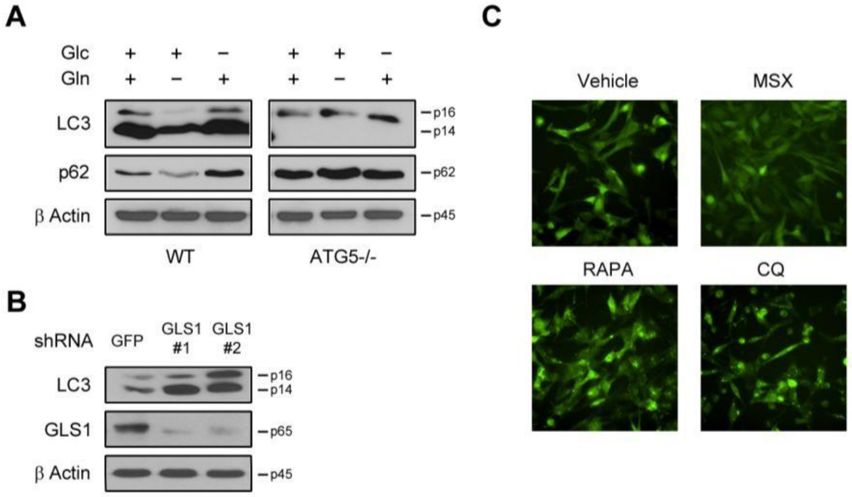

Supplementary Figure 1. (A) WT MEFs and *atg5*-/- MEFs expressing KRas G12V were plated in the complete medium, which was replaced by glucose or glutamine-free medium the following day and then incubated for another 24 h. Cell lysates were immunoblotted for LC3 and p62. (B) Effect of GLS1 knockdown on LC3 levels in MIAPaCa2 cells expressing a control (shGFP) or glutaminase 1 shRNAs (shGLS1s). (C) MIAPaCa2 cells were infected with a retrovirus expressing GFP-LC3, grown in complete, in the treatment with L-Methionine sulfoximine (MSX; 100 μM), rapamycin (RAPA; 1 μM) or chloroquine (CQ; 50 μM) for 18 h and analyzed for formation of LC3 dots to monitor autophagy activity by fluorescence microscopy.

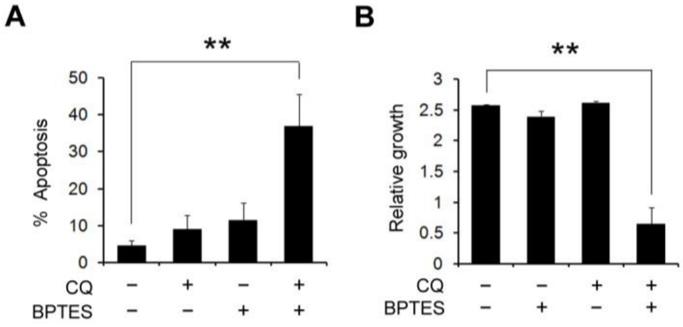

Supplementary Figure 2. (A) MIAPaCa2 cells were treated with CQ (10 μM) and BPTES (10 μM) alone or in combination for 24 h and cell death was assessed by using the annexin V/PI assay. (B) MIAPaCa2 cells were treated with CQ (10 μM) and BPTES (25 μM) alone or in combination. After 72 h-incubation, cellular growth was assessed by using an MTT assay. Error bars represent the s.d. of three separate experiments. **, $p < 0.01$.

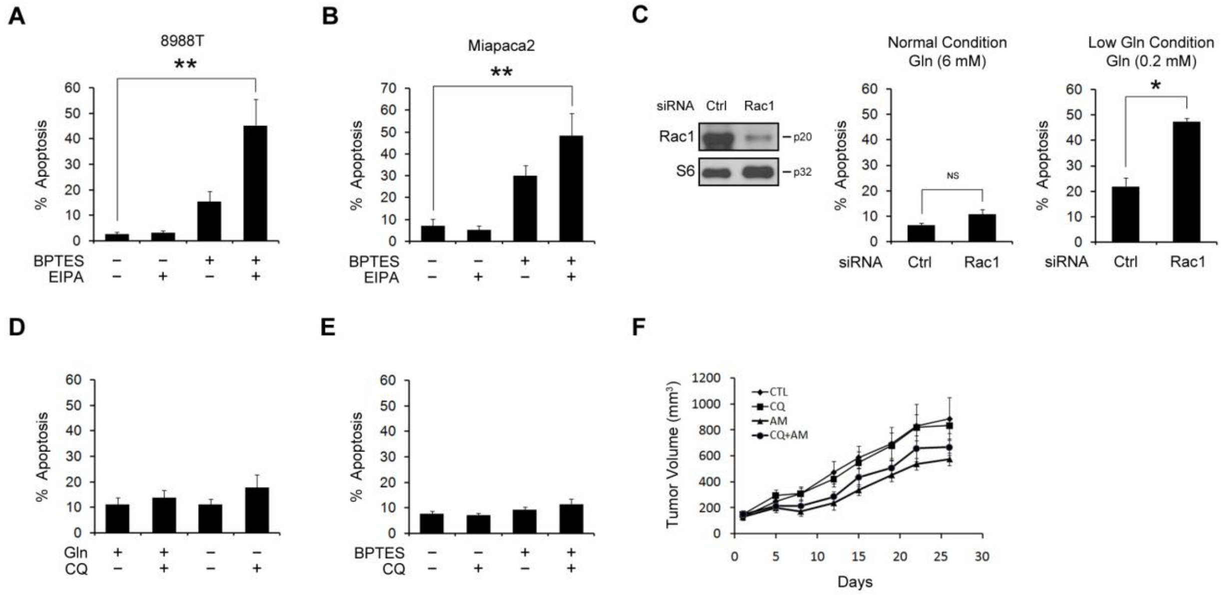

Supplementary Figure 3. (A and B) PDAC cells were treated with BPTES (10 μM) and EIPA (25 μM) alone or in combination for 24 h and cell death was assessed by the annexin V/PI assay. (C) MIAPaCa-2 cells were plated in the complete medium with 50nM Rac1 siRNA, which was replaced with low glutamine medium (0.2mM glutamine) the following day and then incubated for another 48 h. Cell death was assessed by using the annexin V/PI assay. (D) BXPC3 cells were plated in the complete medium, which was replaced with glutamine-free medium the following day and then incubated for another 24 h with or without CQ (10 μM). Cell death was assessed by using the annexin V/PI assay. (E) BXPC3 cells were treated with CQ (10 μM) and BPTES (10 μM) alone or in combination for 24 h and cell death was assessed by using the annexin V/PI assay. (F) Concomitant treatment with CQ and amiloride did not exhibit significant inhibition of tumor growth in a xenograft mouse model. Subcutaneous MIA PaCa-2-driven tumors were established in 6-week old male mice. CQ (20 mg kg^{-1} per day), or amiloride (10 mg kg^{-1} per day) alone, or in combination, were administered daily via intraperitoneal injection. Tumor growth was assessed once tumor volume reached 150 mm^3. Data are shown as the mean of five mice in each group +/−SEM. *, $p < 0.05$; **, $p < 0.01$.

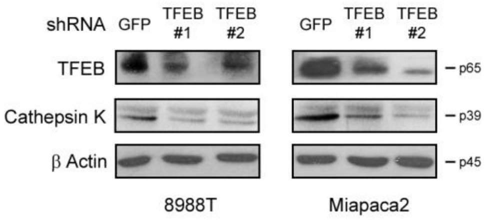

Supplementary Figure 4. Effect of TFEB knockdown on Cathepsin K levels in PDAC cells expressing a control shRNA (shGFP) or TFEB shRNAs.

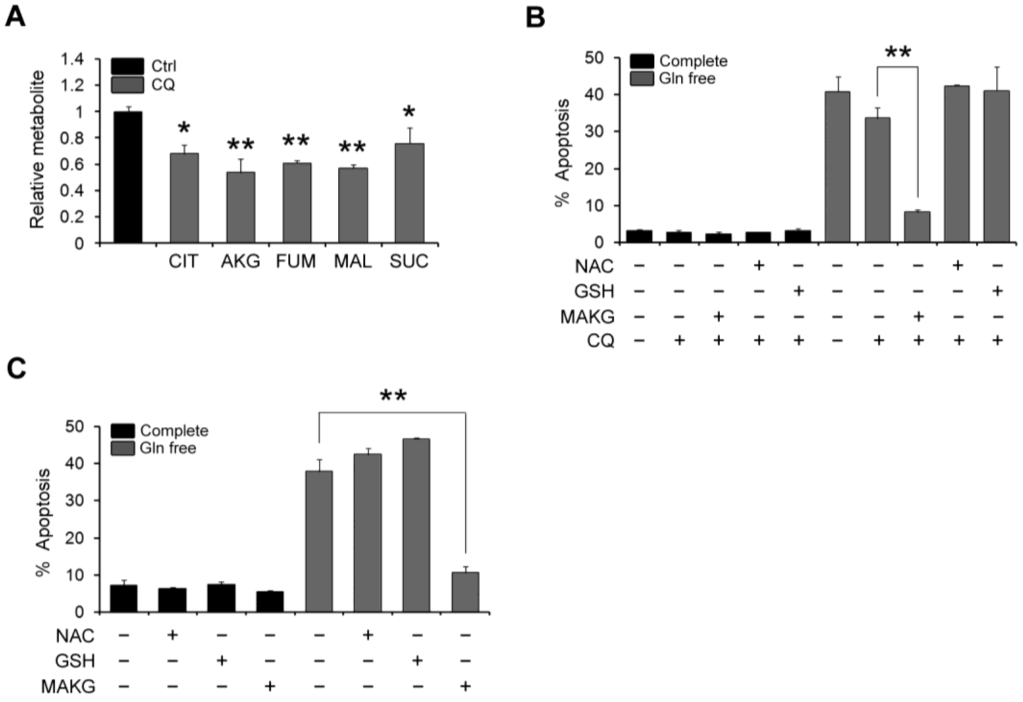

Supplementary Figure 5. (A) TCA metabolite pools were analyzed by using LC-MS/MS in 8988T cells treated with CQ (10 μM) for 24 h. Error bars represent the s.d. of triplicate wells from a representative experiment. (B and C) WT MEFs (B) and *atg5*-/- MEFs (C) expressing KRas G12V were plated in the complete medium which was replaced by glutamine-free medium supplemented with NAC (4 mM), GSH (4 mM), or MAKG (8 mM) the following day and then incubated for another 24 h. Cell death was assessed by the annexin V/PI assay. Error bars represent the s.d. of three separate experiments. *, $p < 0.05$; **, $p < 0.01$.

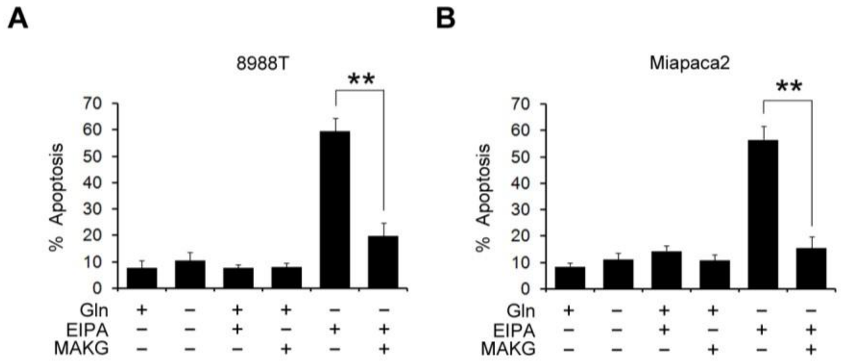

Supplementary Figure 6. (A and B) PDAC cells were plated in the complete medium, which was replaced by glutamine-free medium supplemented with MAKG (2 mM) in the absence or presence of EIPA (25 μM) the following day and then incubated for another 24 h. Cell death was assessed by using the annexin V/PI assay. Error bars represent the s.d. of three separate experiments. **, $p < 0.01$.
